# Discordance between patient and physician global assessment of disease activity in Behçet’s syndrome: a multicenter study cohort

**DOI:** 10.1186/s13075-020-02362-1

**Published:** 2020-11-25

**Authors:** Alberto Floris, Gerard Espinosa, Luisa Serpa Pinto, Nikolaos Kougkas, Andrea Lo Monaco, Giuseppe Lopalco, Ida Orlando, George Bertsias, Luca Cantarini, Ricard Cervera, João Correia, Marcello Govoni, Florenzo Iannone, Alessandro Mathieu, Piergiorgio Neri, Ana Martins Silva, Carlos Vasconcelos, Monica Muntoni, Alberto Cauli, Matteo Piga, the BODI Project collaborators, Nestor Avgoustidis, Ignazio Cangemi, Elisabetta Chessa, Mattia Congia, Maria Ester D’Amico, Raquel Faria, Gema M. Lledó, Vittorio Pirani, Roberto Ríos-Garcés, Ernestina Santos, Vincenzo Venerito, Antonio Vitale

**Affiliations:** 1 Rheumatology Unit, AOU University Clinic, Cagliari, Italy; 2 Dipartimento di Scienze Mediche e Sanità Pubblica, Università di Cagliari, SS 554, 09042 Monserrato, Cagliari Italy; 3 Department of Autoimmune Diseases, Hospital Clinic, Institut d’Investigacions Biomediques August Pi i Sunyer (IDIBAPS), University of Barcelona, Barcelona, Catalonia Spain; 4 Hospital Santo Antonio Centro Hospitalar do Porto, Unidade de Imunologia Clinica, Porto, Portugal; 5 Rheumatology, Clinical Immunology and Allergy Unit, University of Crete, Heraklion, Greece; 6 Rheumatology Unit, AOU S. Anna di Ferrara, University of Ferrara, Ferrara, Italy; 7 Rheumatology Unit, University of Bari, Bari, Italy; 8 Rheumatology Unit, University of Siena, Siena, Italy; 9 Ophthalmology Clinic, Università Politecnica delle Marche, Ancona, Italy; 10 Cleveland Clinic Abu Dhabi, Eye Institute, Abu Dhabi, United Arab Emirates; 11 Neurology Department, Centro Hospitalar do Porto/Hospital de Santo António, Porto, Portugal; 12 UMIB Abel Salazar Biomedical Sciences Institute, University of Porto, Porto, Portugal; 13 Associazione Italiana Sindrome e Malattia di Behçet (SIMBA), Pontedera, Italy

**Keywords:** Behçet’s syndrome, Disease activity, Outcome measure, Patients reported outcomes

## Abstract

**Background:**

To compare the patients’ and physician’s global assessment of disease activity in Behçet’s syndrome (BS) and investigate the frequency, magnitude, and determinants of potential discordance.

**Methods:**

A total of 226 adult BS patients with a median (IQR) age of 46.9 (35.6–55.2) years were enrolled across Italy, Greece, Portugal, and Spain. Demographic, clinical, and therapeutic variables, as well as the patient reported outcomes, were collected at the recruitment visit. The physical (PCS) and mental (MCS) component summary scores of the Short Form Questionnaire 36 (SF-36) and the Behçet’s syndrome Overall Damage Index (BODI) were calculated. Disease activity was assessed by the patients’ (PtGA) and physician’s global assessment (PGA) in a 10-cm visual analog scale, as well as the Behçet Disease Current Activity Form (BDCAF). Discordance (∆) was calculated by subtracting the PGA from the PtGA and defined as positive (PtGA>PGA) and negative (PtGA<PGA) discordance using both a more stringent (∆ = ±2) and a less stringent (∆ = ±1) cutoff. Univariate and multivariate logistic regressions were performed.

**Results:**

Median PtGA and PGA scores were 2.0 (0.3–5.0) and 1.0 (0.0–3.0) cm, respectively. The discordance prevalence varied (from 29.6 to 55.3%) according to the cutoff applied, and the majority (> 80%) of disagreements were due to patients rating higher their disease activity. Higher values of BDCAF were associated to increased rate of positive discordance. When BDCAF = 0, the median (IQR) values of PtGA and PGA were 0.2 (0–2) and 0 (0–1), respectively. PCS (adjusted odds ratio (adjOR) 0.96 per unit, 95% CI 0.93–0.98, *p* = 0.006) and MCS (adjOR 0.96 per unit, 95% CI 0.93–0.99, *p* = 0.003) were independently associated with positive discordance using both cutoffs. Active ocular involvement emerged as a potential determinant of negative discordance (adjOR 5.88, 95% CI 1.48–23.30, *p* = 0.012).

**Conclusions:**

PtGA and PGA should be considered as complementary measures in BS, as patients and physicians may be influenced by different factors when assessing active disease manifestations. Particularly, PtGA may be a useful tool in the assessment of BS disease activity, as it carries a low risk to misclassify an inactive disease, and may allow to capture aspects of the patient’s health that negatively affect his well-being and the treatment.

**Supplementary information:**

**Supplementary information** accompanies this paper at 10.1186/s13075-020-02362-1.

## Introduction

Behçet’s syndrome (BS) is a multisystem inflammatory disease of unknown etiology, characterized by strong genetic background, distinctive geographic distribution, and a wide variability in clinical presentation [1–3]. It typically manifests with oral and genital ulcers, skin lesions, and uveitis, but musculoskeletal, nervous, vascular, and gastrointestinal involvement may occur leading to significant morbidity and mortality [4].

Because of its clinical variability, it is difficult to define the disease activity in BS, and a validated and widely accepted tool for its measurement is not yet available.

The patient’s global assessment (PtGA) and physician’s global assessment (PGA) of disease activity on visual analogic scales have been frequently used in studies involving patients with BS. Indeed, although not formally validated, both PtGA and PGA have been endorsed for inclusion into the OMERACT core set of outcome measures for BS [5].

According to the European League Against Rheumatism (EULAR) recommendations, the treatment of BS should be individually tailored and therapeutic decisions should be based on a consultation between patient and physician through shared decision-making [6]. Indeed, many studies on several chronic illnesses have shown that, when there is concordance between patients’ and physicians’ judgment on the disease status, adherence to treatment and outcomes significantly improve [7–9].

However, the patients’ opinions on disease activity do not always match those of their physicians and frequent discordance has been demonstrated in several rheumatic diseases including rheumatoid arthritis [10–12], psoriatic arthritis [13, 14], ankylosing spondylitis [15], and systemic lupus erythematosus [16]. Despite its potential clinical relevance, little is known so far about the frequency, magnitude, and potential determinants of discordance between patients and physicians in BS.

In the view of an enhanced patient-physician partnership in the disease management, this study aimed to investigate the frequency, magnitude, and determinants of discordance in the patients’ and physician’s global assessment of disease activity in a multicenter cohort of BS patients.

## Methods

### Patients

A post hoc analysis on data from patients recruited in the BODI Project validation cohort was performed. The BODI Project is an international multicenter cross-sectional study aiming to develop and preliminarily validate the Behçet’s syndrome Overall Damage Index (BODI), a tool specifically designed to identify and measure organ damage in BS [17]. The BODI validation cohort consisted of 228 adult patients (≥ 18 years old), diagnosed with BS according to the ISG [18] or ICBD [19] criteria, and having a disease duration of at least 12 months. The study was conducted according to general and local regulation and approved by the ethics committee of the coordinating center at the AOU of Cagliari (Prot. PG/2018/17158).

### Data collection

Demographic, clinical, and therapeutic data were assessed and recorded by the recruiting physician at the enrolment visit. Self-reported outcomes were recorded by the patients during the same visit. Active disease manifestations were categorized as follows: mucocutaneus (oral aphthosis and/or genital ulcers and/or skin lesions), arthritis, ocular, and major organ involvement (vascular and/or GI and/or nervous lesions). The presence of any chronic comorbidity was also recorded (malignancy, cardiovascular and respiratory disease, metabolic disorders, major depressive disorder, other immunomediate diseases). Disease activity was evaluated by the PtGA and PGA, as well as the Behçet Disease Current Activity Form (BDCAF) [20]. PtGA and PGA were assessed through a single question (“How active was your/the patient’s BS during the last week?”) on an anchored 10-cm visual analogic (0.5-cm graded), where 0 corresponded to “no disease activity” and 10 to “the highest disease activity.” At each center, the PGA assessor was a single physician with expertise in BS. Irreversible organ damage accrual was also assessed by using the BODI [17]. Different aspects of the health-related quality of life (HR-QoL) were assessed through the patient-rated Short Form Questionnaire in 36 items (SF-36), and the physical (PCS) and mental (MCS) component summary scores were recorded [21]. Finally, the ongoing therapies at the enrolment visit were recorded.

### Definition of discordance

**Fig. 1:**
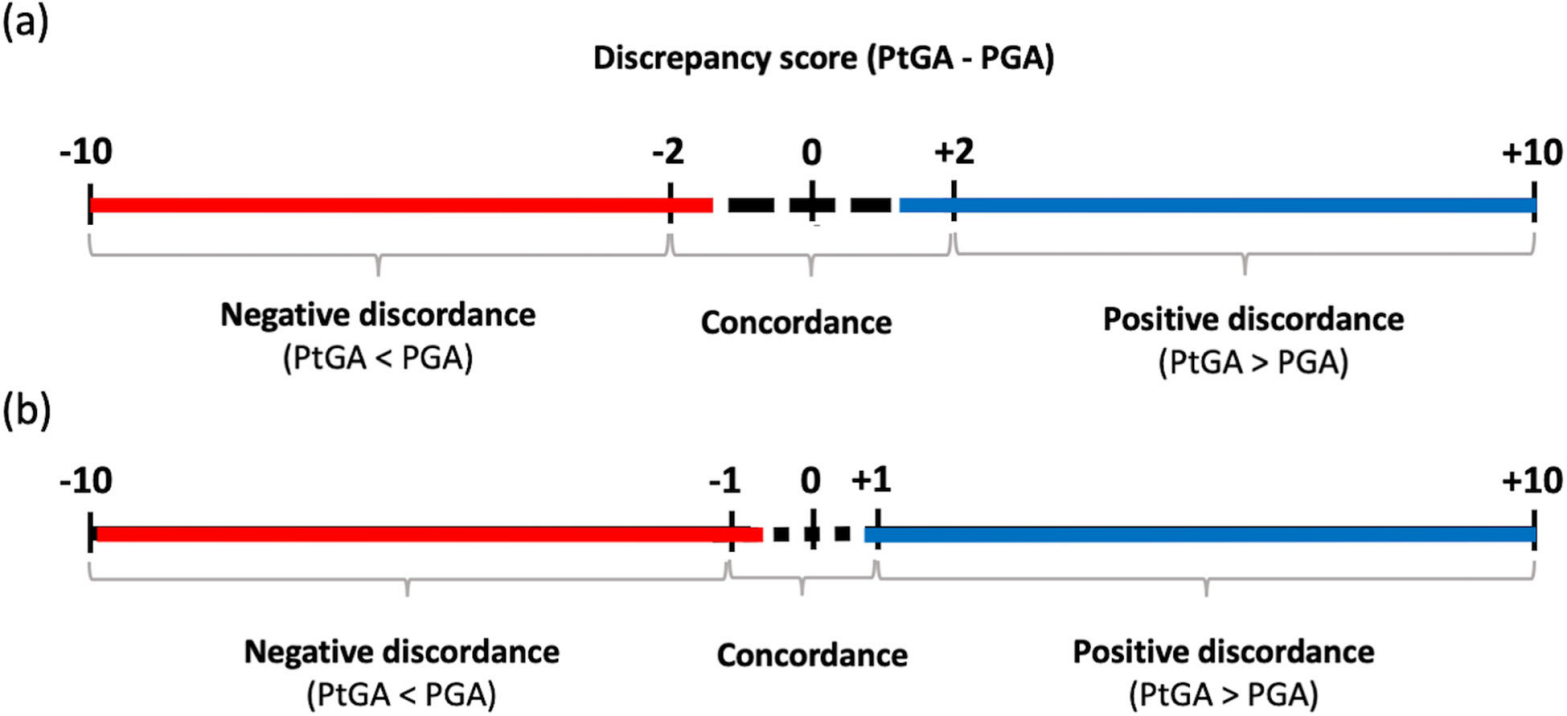
Categorization of PtGA-PGA discordance using a cutoff of (**a**) ± 2 and (**b**) ± 1. PtGA, patient global assessment of disease activity; PGA, physician global assessment of disease activity

A discrepancy score was calculated by subtracting the PGA from the PtGA (“PtGA − PGA”). As there was not a validated definition of clinically relevant difference between such measures, two cutoffs, one more stringent (± 2) and one less stringent (± 1), were chosen by rounding to the standard deviation of the mean absolute discrepancy score in the BODI cohort. Thus, the relationship between PtGA and PGA was classified in three categories: (a) positive discordance, when the patient rated higher than her/his physician (“PtGA − PGA” ≥ + 2 or + 1); (b) negative discordance, when the patient rated lower than her/his physician (“PtGA − PGA” ≤ − 2 or − 1); and (c) concordance, when the discrepancy score was > − 2 and < + 2 or > − 1 and < + 1 (Fig. 1).

To assess the potential impact of the extent of disease activity, when assessed by an objective instrument, the rate of discordance/concordance was assessed by stratifying patients according to different ranges of the corrected BDCAF score (0, 1–3, 4–6, and ≥ 7 points).

### Candidate determinants of discordance

Age, gender, disease duration, chronic comorbidities, active disease manifestations, and ongoing treatments were explored as potential demographic, clinical, and therapeutic determinants of discordance between PtGA and PGA. To investigate the potential misleading impact of damage accrual in the overall assessment of disease activity, correlation between BODI score and the PtGA-PGA discordance was also analyzed. To explore the potential influence of different aspects of the patient’s mental and physical domains of the HR-QoL, the PCS and MCS scales of the SF-36 were analyzed as potential determinants of discrepancy.

### Statistical analysis

The sample distribution of variables was described as mean ± standard deviation (SD) or median with interquartile range (IQR), for continuous variables, or as frequencies and percentages, for categorical variables.

The discrepancy score was calculated, and the prevalence of positive and negative discordance was separately evaluated by using both the thresholds of ± 2 and ± 1.

The association between PtGA-PGA discordance (dependent variable) and its potential determinants (independent variables) was evaluated through univariate and multivariate logistic regression, and results were presented as odds ratio (OR) with 95% CI both crude and adjusted (adj). Variables showing associations with *p* values ≤ 0.10 in univariate analysis were included in multivariate analysis. Both for positive and negative discordance, two separate analyses have been performed by using the cutoff of ± 2 and ± 1 for significant discrepancy.

The statistical significance was set for *p* < 0.05. Statistical analyses were performed using SPSS® software (version 24.0, Armonk, NY, USA).

## Results

### Patients

Out of the 228 patients enrolled in the BODI cohort, 226 were recruited for the present study. Two were excluded for missing PtGA or PGA data. Males were 111 (49.1%). The median (IQR) enrollment age and disease duration were 46.9 (35.6–55.2) and 11.7 (5.9–20.8) years, respectively. The median (IQR) BDCAF score was 3.0 (0.0–5.0), with 106 (46.9%) patients having at least one active disease manifestation. Details on the baseline characteristic of the studied cohort are reported in Table 1.

**Table 1:** Baseline features of the study cohort (*n* = 226)

**Males,** ***n*** **(%)**	111 (49.1%)
**Age at enrolment, median (IQR) years**	46.9 (35.6–55.2)
**Disease duration, median (IQR) years**	11.7 (5.9–20.8)
**Comorbidities,** ***n*** **(%)**	99 (43.8%)
**Active disease manifestations**	
Mucocutaneous lesions, *n* (%)	83 (36.7%)
Ocular involvement, *n* (%)	12 (5.3%)
Arthritis, *n* (%)	17 (7.5%)
Major organ involvement, *n* (%)	20 (8.8%)
**Disease activity indices**	
PGA, median (IQR) score	1.0 (0.0–3.0)
PtGA, median (IQR) score	2.0 (0.3–5.0)
BDCAF, median (IQR) score	3.0 (0.0–5.0)
**Irreversible organ damage**	
BODI, median (IQR) score	1.0 (0.0–2.0)
**HR-QoL (by SF-26)**	
PCS, median (IQR) score	46.6 (38.8–54.2)
MCS, median (IQR) score	44.9 (35.9–54.4)
**Current and past therapy**	
Colchicine, *n* (%)	107 (47.3%)
Glucocorticoids, *n* (%)	104 (45.6%)
Immunosuppressants, *n* (%)	95 (42.0%)
Biologics, *n* (%)	49 (21.7%)

*IQR* interquartile range, *BDCAF* Behçet’s Disease Current Activity Form, *PGA* physician global assessment, *PtGA* patient global assessment of disease activity, *BODI* Behçet’s syndrome Overall Damage Index, *HR-QoL* health-related quality of life, *PCS* physical component summary in the SF-36 questionnaire, *MCS* mental component summary in the SF-36 questionnaire

### Prevalence and magnitude of discordance

In the whole cohort, the median (IQR) PtGA and PGA scores were respectively 2.0 (0.3–5.0) and 1.0 (0.0–3.0) cm, with a mean (± SD) absolute discrepancy score of 1.3 (1.5).

**Fig. 2:**
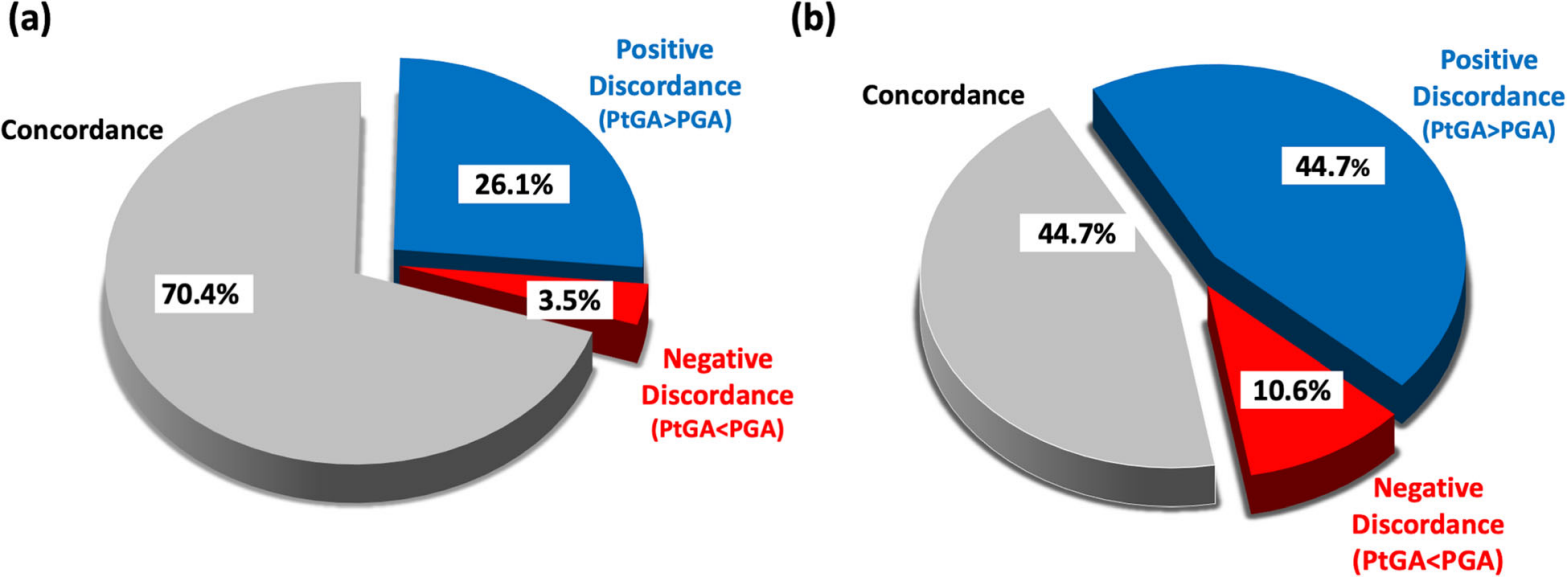
Frequency of positive and negative discordance and concordance in the patients’ (PtGA) and physicians’ global assessment (PGA) of disease activity, by using a PtGA-PGA discrepancy score of ± 2 (**a**) and ± 1 (**b**) as a threshold for significant discordance

When the discordance between PtGA and PGA was analyzed by using a discrepancy score threshold of ± 2, positive and negative discordance were recorded in 59 (26.1%) and 8 (3.5%) patients, respectively, whereas concordance was observed in the remaining 159 (70.4%) cases (Fig. 2a). When a discrepancy score of ± 1 was applied, positive discordance, negative discordance, and concordance rates were 101 (44.7%), 24 (10.6%), and 101 (44.7%) patients, respectively (Fig. 2b).

**Fig. 3:**
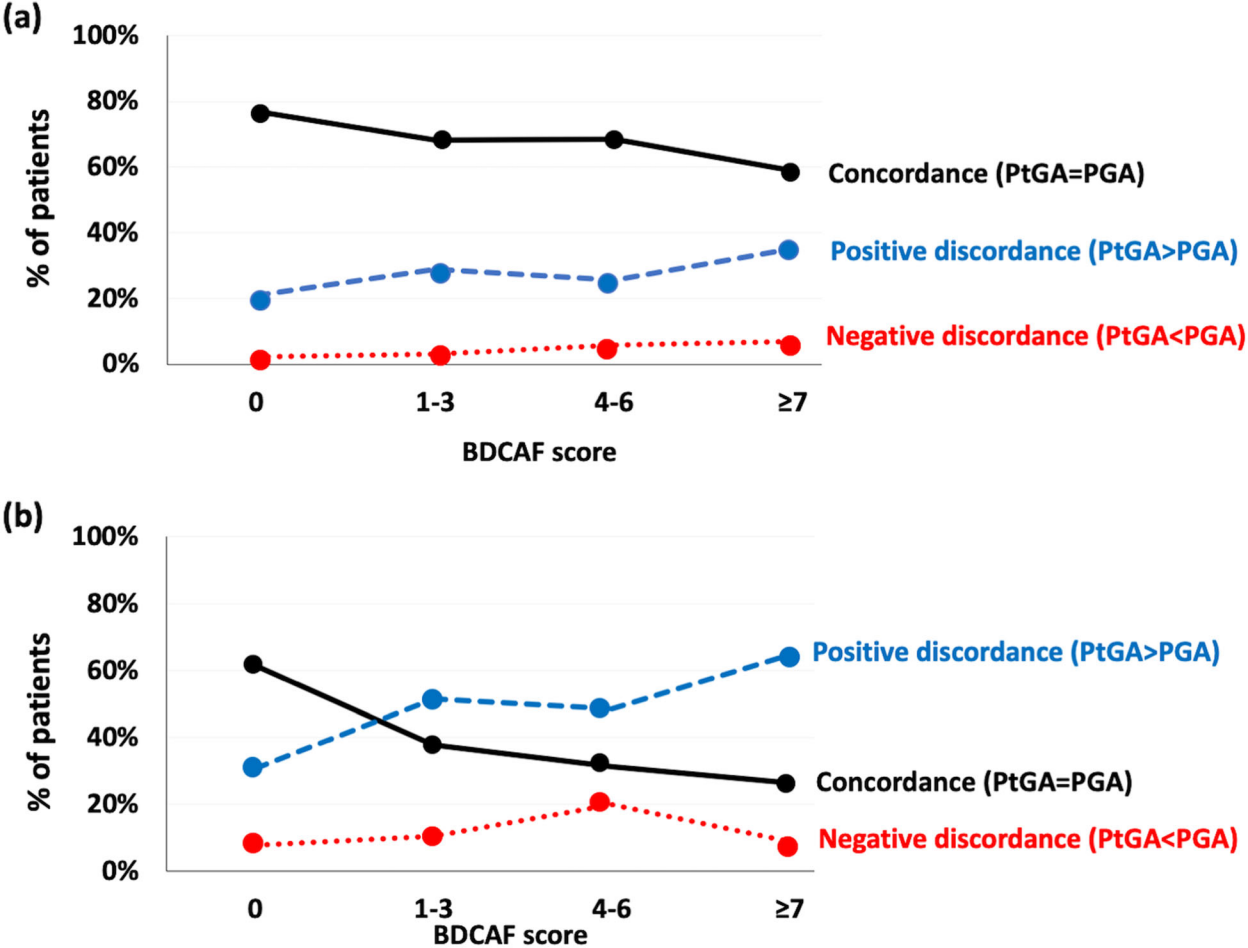
Changes in the prevalence of the PtGA-PGA discordance according to the different levels of disease activity, as assessed by the BDCAF. **a** A cutoff of ± 1 was applied; **b** ± 2. PtGA, patient’s global assessment; PGA, physician’s global assessment; BDCAF, Behçet’s Disease Current Activity Form

When patients were stratified according to the BDCAF score, a progressive decrease in the frequency of concordance and a concomitant increase in the frequency of positive discordance were recorded, both using the cutoff of ± 2 and, more remarkably, of ± 1. Negative discordance rate remained substantially stable (Fig. 3). In patients with BDCAF = 0, the median (IQR) value of PtGA and PGA was 0.2 (0–2) and 0 (0–1), respectively.

### Determinants of positive discordance (PtGA>PGA)

In univariate analysis, positive discordance, as defined by a discrepancy score ≥ + 2, was significantly associated with ongoing treatment with glucocorticoids (OR 2.07, 95% CI 1.13–3.79, *p* 0.018) and lower scores in the PCS (OR 0.95, 95% CI 0.92–0.97, *p* < 0.001) and MCS (OR 0.95, 95% CI 0.93–0.98, *p* < 0.001) scales of the SF-36 questionnaire. In multivariate analysis, only PCS (adjusted odds ratio (adjOR) 0.96, 95% CI 0.93–0.98, *p* = 0.006) and MCS (adjOR 0.96, 95% CI 0.93–0.99, *p* = 0.003) were independently associated with positive discordance (Supplementary material).

When a discrepancy score ≥ + 1 was applied to identify positive discordance, a significant association in univariate analysis was found for active mucocutaneous lesions (OR 1.99, 95% CI 1.15–3.44, *p* = 0.014), ongoing treatment with glucocorticoid (OR 2.00, 95% CI 1.17–3.40, *p* = 0.011), and lower values of the PCS (OR 0.95, 95% CI 0.93–0.98, *p* < 0.001) and MCS (OR 0.94, 95% CI 0.92–0.96, *p* < 0.001). In multivariate analysis, an independent association was confirmed only for PCS (adjOR 0.97, 95% CI 0.92–1.00, *p* = 0.046) and MCS (adjOR 0.95, 95% CI 0.92–0.98, *p* < 0.001) (Supplementary material).

### Determinants of negative discordance (PtGA<PGA)

In univariate analysis, negative discordance, as defined by a discrepancy score ≤ − 2, was significantly associated with active ocular involvement (OR 6.93, 95% CI 1.24–38.78, *p* = 0.027), whereas a trend toward statistical significance was found for the association with the presence of any chronic comorbidity (OR 4.03, 95% CI 0.80–20.43, *p* = 0.092). In multivariable analysis, active ocular lesions were confirmed to be the only factor independently associated with negative discordance (adjOR 7.68, 95% CI 1.29–45.59, *p* = 0.025) (Supplementary material).

When a discrepancy score ≤ − 1 was applied to identify negative discordance, a significant association in univariate analysis was found for active ocular involvement (OR 4.85, 95% CI 1.34–17.54, *p* = 0.016), whereas a trend toward statistical significance was found for the presence of any chronic comorbidity (OR 2.34, 95% CI 0.98–5.60, *p* = 0.056) and not-use of biologic drugs (OR 0.14, 95% CI 0.02–1.06, *p* = 0.057). In multivariable analysis, active ocular lesions were confirmed to be the only factor independently associated with negative discordance (adjOR 5.88, 95% CI 1.48–23.30, *p* = 0.012) (Supplementary material).

## Discussion

This study provides original and meaningful data on the frequency, extent, and potential determinants of the gap between patient’s and physician’s assessment of disease activity in BS.

In our multicenter cohort, PtGA was on average higher than PGA and the prevalence of discordance varied (from 29.6 to 55.3%) according to the cutoff applied for its definition. In case of disagreement, most patients rated higher disease activity than their physicians (> 80%), whereas negative discordance was rare. Further, discordance was more frequent in patients with high disease activity, according to the BDCAF. The patient-perceived physical and mental domains of QoL played a major role in the occurrence of positive PtGA-PGA discordance. Ocular involvement emerged as a potential determinant of negative discordance, even though the reliability of such result may be affected by the low of prevalence of this type of discordance and the small number of cases with active eye involvement.

The data on prevalence of PtGA-PGA discordance we recorded in our BS cohort are consistent with those reported in several other studies on different rheumatic diseases, where a trend toward a higher rating of disease activity by patients than by their physicians was observed in 30–50% of cases. In many of these studies, the authors concluded that using PtGA without an adequate counterbalance by other objective indices may result in overtreatment of patients because of non-inflammatory alterations that are unresponsive to the pharmacological therapy [7–11]. In our cohort, we observed a high frequency of agreement between PtGA and PGA in patients with low BDCAF values, whereas the level of disagreement grew up as the BDCAF values increased. These findings suggest that patients and physicians have a higher level of agreement in judging inactive disease state, whereas they mainly disagree in judging the extent and severity of active manifestations. Moreover, evidence of the self-perceived physical and mental domains of QoL as main determinant of discordance would reveal that, when patients are asked to evaluate how much their BS is active, they might be influenced by other aspects of their health and well-being. From a clinical point of view, the agreement between PtGA and PGA in scoring low levels of disease activity ensures a high probability of correctly classifying the low BS activity state. This could be of special interest for the future definition of core set domains for low disease activity or disease remission. On the other hand, disagreement in judging the extent of disease activity should induce clinicians to an in-depth assessment aimed to identify the reasons for discordance and exclude incipient or unapparent disease manifestations. Further longitudinal studies are needed to understand how considering the PtGA-PGA disagreement in the therapeutic decision-making.

Some limitations should be taken into account interpreting the results of our study. First, the small number of patients from other countries with high prevalence of BS may limit the generalizability of our findings. Second, education and socio-economic factors were not included as potential determinants of discordance, because of the lack of these data in the BODI study. Finally, the low frequency of active major organ involvement in our cohort may have prevented to identify its role in explaining the occurrence of PtGA-PGA discordance.

## Conclusions

Patient-assessed and physician-assessed measures of disease activity should be considered as complementary and should be used together to improve the mutual understanding of disease activity state, as well as the partnership in the disease management. Particularly, PtGA may be a useful tool in the assessment of BS disease activity, as it carries a low risk to misclassify an inactive disease, and may allow to capture aspects of the patients health that negatively affect their well-being and the treatment outcomes. Further research is needed to evaluate the long-term consequence of discordance and the benefit of specific interventions for its reduction.

## Supplementary Information

**Additional file 1: Table S1.** Determinants of positive discordance (PtGA > PGA) with a discrepancy threshold of ≥ +2. **Table S2.** Determinants of positive discordance (PtGA > PGA) with a discrepancy threshold ≥ +1. **Table S3.** Determinants of negative discordance (PtGA < PGA) with a discrepancy threshold of ≤ -2. **Table S4.** Determinants of negative discordance (PtGA < PGA) with a discrepancy threshold of ≤ -1.

## Data Availability

The datasets analyzed during the current study are available from the corresponding author on reasonable request.

## Abbreviations

BS: Behçet’s syndrome
PtGA: Patient’s global assessment
PGA: Physician’s global assessment
OMERACT: Outcome Measures in Rheumatology Clinical Trials
EULAR: European League Against Rheumatism
BODI: Behçet’s syndrome Overall Damage Index
ISG: International Study Group for Behçet’s Disease
ICBD: International Criteria for Behçet’s Disease
BDCAF: The Behçet Disease Current Activity Form
HR-QoL: Health-related quality of life
SF36: Short Form Questionnaire in 36 items
PCS: Physical component summary
MCS: Mental component summary
SD: Standard deviation
IQR: Interquartile range
OR: Odds ratio
adjOR: Adjusted odds ratio
CI: Confidence interval
DAS28: Disease Activity Score on 28 Joints
