# Supplementary material for: Discordance between patient and physician global assessment of disease activity in Behçet’s syndrome: a multicenter study cohort
Source: Arthritis Res Ther. 2020 Nov 25;22:278. doi: 10.1186/s13075-020-02362-1 (PMC7687797; doi:10.1186/s13075-020-02362-1)
Supplement: Supplementary file 1 — Additional file 1: Table S1. Determinants of positive discordance (PtGA > PGA) with a discrepancy threshold of ≥ +2. Table S2. Determinants of positive discordance (PtGA > PGA) with a discrepancy threshold ≥ +1. Table S3. Determinants of negative discordance (PtGA < PGA) with a discrepancy threshold of ≤ -2. Table S4. Determinants of negative discordance (PtGA < PGA) with a discrepancy threshold of ≤ -1. [file 13075_2020_2362_MOESM1_ESM.docx]

| **Supplementary table 1.** Determinants of positive discordance (PtGA > PGA) with a discrepancy threshold of ≥ +2 | | | | | |
| --- | --- | --- | --- | --- | --- |
|  | Univariate analysis | |  | Multivariable analysis | |
|  | OR (95%CI) | p |  | adjOR (95%CI) | p |
| **Demographics** |  |  |  |  |  |
| Male | 0.63 (0.35-1.15) | 0.133 |  |  |  |
| Age at enrolment | 1.00 (0.98-1.03) | 0.327 |  |  |  |
| Disease duration | 1.00 (0.97-1.03) | 0.785 |  |  |  |
| Comorbidities | 0.84 (0.46-1.54) | 0.573 |  |  |  |
| **Active manifestations** |  |  |  |  |  |
| Mucocutaneous | 1.03 (0.56-1.91) | 0.917 |  |  |  |
| Ocular | 0.94 (0.25-3.60) | 0.929 |  |  |  |
| Major organ involvement | 1.24 (0.45-3.39) | 0.678 |  |  |  |
| Arthritis | 1.20 (0.40-3.56) | 0.747 |  |  |  |
| **Organ Damage** |  |  |  |  |  |
| BODI score | 0.96 (0.82-1.11) | 0.550 |  |  |  |
| **HR-QoL** |  |  |  |  |  |
| PCS | 0.95 (0.92-0.97) | **<0.001** |  | 0.96 (0.93-0.99) | **0.006** |
| MCS | 0.95 (0.93-0.98) | **<0.001** |  | 0.96 (0.93-0.99) | **0.003** |
| **Ongoing treatment** |  |  |  |  |  |
| Glucocorticoids | 2.07 (1.13-3.79) | **0.018** |  | - | - |
| Colchicine | 0.92 (0.51-1.66) | 0.777 |  |  |  |
| Immunosuppressants | 1.23 (0.68-2.23) | 0.500 |  |  |  |
| Biologics | 1.33 (0.66-2.68) | 0.418 |  |  |  |
| PtGA, patient’s global assessment. PGA, physician global assessment. BODI, Behçet’s syndrome overall damage index. PCS, physical component summary in SF-36 questionnaire. MCS, physical component summary in SF-36 questionnaire. | | | | | |

| **Supplementary table 2.** Determinants of positive discordance (PtGA > PGA) with a discrepancy threshold ≥ +1. | | | | | |
| --- | --- | --- | --- | --- | --- |
|  | Univariate analysis | |  | Multivariable analysis | |
|  | OR (95%CI) | p |  | adjOR (95%CI) | p |
| **Demographics** |  |  |  |  |  |
| Male | 0.77 (0.46-1.31) | 0.335 |  |  |  |
| Age at enrolment | 1.00 (0.98-1.02) | 0.757 |  |  |  |
| Disease duration | 0.99 (0.96-1.01) | 0.360 |  |  |  |
| Comorbities | 0.79 (0.46-1.34) | 0.382 |  |  |  |
| **Active manifestations** |  |  |  |  |  |
| Mucocutaneous | 1.99 (1.15-3.44) | **0.014** |  |  |  |
| Ocular | 0.60 (0.18-2.06) | 0.420 |  |  |  |
| Major organ involvement | 0.81 (0.32-2-07) | 0.659 |  |  |  |
| Arthritis | 1.43 (0.53-3.85) | 0.479 |  |  |  |
| **Organ Damage** |  |  |  |  |  |
| BODI score | 0.93 (0.82-1.06) | 0.291 |  |  |  |
| **HR-QoL** |  |  |  |  |  |
| PCS | 0.95 (0.93-0.98) | **<0.001** |  | 0.97 (0.92-1.00) | **0.046** |
| MCS | 0.94 (0.92-0.96) | **<0.001** |  | 0.95 (0.92-0.97) | **<0.001** |
| **Ongoing treatment** |  |  |  |  |  |
| Glucocorticoids | 2.00 (1.17-3.40) | **0.011** |  |  |  |
| Colchicine | 1.01 (0.60-1.71) | 0.961 |  |  |  |
| Immunosuppressants | 1.20 (0.71-2.05) | 0.491 |  |  |  |
| Biologics | 1.25 (0.661-2.35) | 0.495 |  |  |  |
| PtGA, patient’s global assessment. PGA, physician global assessment. BODI, Behçet’s syndrome overall damage index. PCS, physical component summary in SF-36 questionnaire. MCS, physical component summary in SF-36 questionnaire. | | | | | |

| **Supplementary table 3.** Determinants of negative discordance (PtGA < PGA) with a discrepancy threshold of ≤ -2 | | | | | |
| --- | --- | --- | --- | --- | --- |
|  | Univariate analysis | |  | Multivariable analysis | |
|  | OR (95%CI) | p |  | adjOR (95%CI) | p |
| **Demographics** |  |  |  |  |  |
| Male | 0.00 (0.00-0.00) | 0.998 |  |  |  |
| Age at enrolment | 1.03 (0.97-1.09) | 0.358 |  |  |  |
| Disease duration | 1.02 (0.95-1.09) | 0.594 |  |  |  |
| Comorbidities | 4.03 (0.80-20.43) | **0.092** |  |  |  |
| **Active manifestations** |  |  |  |  |  |
| Mucocutaneous | 1.76 (0.43-7.23) | 0.433 |  |  |  |
| Ocular | 6.93 (1.24-38.78) | **0.027** |  | 7.68 (1.29-45.59) | **0.025** |
| Major organ involvement | 0.00 (0.00-0.00) | 0.996 |  |  |  |
| Arthritis | 0.00 (0.00-0.00) | 0.999 |  |  |  |
| **Organ Damage** |  |  |  |  |  |
| BODI score | 1.08 (0.79-1.47) | 0.624 |  |  |  |
| **HR-QoL** |  |  |  |  |  |
| PCS | 0.95 (0.89-1.01) | 0.126 |  |  |  |
| MCS | 1.04 (0.97-1.11) | 0.286 |  |  |  |
| **Ongoing therapy** |  |  |  |  |  |
| Glucocorticoids | 3.67 (0.73-18.6) | 0.116 |  |  |  |
| Colchicine | 0.36 (0.07-1.82) | 0.215 |  |  |  |
| Immunosuppressants | 1.40 (0.34-5.73) | 0.644 |  |  |  |
| Biologics | 0.51 (0.06-4.24) | 0.529 |  |  |  |
| PtGA, patient’s global assessment. PGA, physician global assessment. BODI, Behçet’s syndrome overall damage index. PCS, physical component summary in SF-36 questionnaire. MCS, physical component summary in SF-36 questionnaire. | | | | | |

| **Supplementary table 4.** Determinants of negative discordance (PtGA < PGA) with a discrepancy threshold of ≤ -1 | | | | | |
| --- | --- | --- | --- | --- | --- |
|  | Univariate analysis | |  | Multivariable analysis | |
|  | OR (95%CI) | p |  | adjOR (95%CI) | p |
| **Demographics** |  |  |  |  |  |
| Male | 0.86 (0.37-2.02) | 0.734 |  |  |  |
| Age at enrolment | 1.03 (0.99-1.07) | 0.099 |  |  |  |
| Disease duration | 1.01 (0.97-1.05) | 0.606 |  |  |  |
| Comorbidities | 2.34 (0.98-5.60) | **0.056** |  | - | - |
| **Active manifestations** |  |  |  |  |  |
| Mucocutaneous | 1.04 (0.43-2.49) | 0.934 |  |  |  |
| Ocular | 4.85 (1.34-17.54) | **0.016** |  | 5.88 (1.48-23.3) | **0.012** |
| Major organ involvement | 0.93 (0.20-4.28) | 0.925 |  |  |  |
| Arthritis | 0.51 (0.6-3.99) | 0.517 |  |  |  |
| **Organ Damage** |  |  |  |  |  |
| BODI score | 1.07 (0.88-1.29) | 0.502 |  |  |  |
| **HR-QoL** |  |  |  |  |  |
| PCS | 0.98 (0.94-1-02) | 0.251 |  |  |  |
| MCS | 1.01 (0.98-1.05) | 0.556 |  |  |  |
| **Ongoing therapy** |  |  |  |  |  |
| Glucocorticoids | 1.44 (0.62-3.37) | 0.399 |  |  |  |
| Colchicine | 0.93 (0.40-2.18) | 0.875 |  |  |  |
| Immunosuppressants | 1.19 (0.51-2.78) | 0.690 |  |  |  |
| Biologics | 0.14 (0.02-1.06) | **0.057** |  | - | - |
| PtGA, patient’s global assessment. PGA, physician global assessment. BODI, Behçet’s syndrome overall damage index. PCS, physical component summary in SF-36 questionnaire. MCS, physical component summary in SF-36 questionnaire. | | | | | |
